# Sexuality after a cancer diagnosis: A population‐based study

**DOI:** 10.1002/cncr.30263

**Published:** 2016-08-16

**Authors:** Sarah E. Jackson, Jane Wardle, Andrew Steptoe, Abigail Fisher

**Affiliations:** ^1^ Department of Epidemiology and Public HealthUniversity College LondonLondonUnited Kingdom

**Keywords:** cancer, sex, sexual activity, sexual concern, sexual function

## Abstract

**BACKGROUND:**

This study explored differences in sexual activity, function, and concerns between cancer survivors and cancer‐free controls in a population‐based study.

**METHODS:**

The data were from 2982 men and 3708 women who were 50 years old or older and were participating in the English Longitudinal Study of Ageing. Sexual well‐being was assessed with the Sexual Relationships and Activities Questionnaire, and cancer diagnoses were self‐reported.

**RESULTS:**

There were no differences between cancer survivors and controls in levels of sexual activity (76.0% vs 78.5% for men and 58.2% vs 55.5% for women) or sexual function. Men and women with cancer diagnoses were more dissatisfied with their sex lives than controls (age‐adjusted percentages: 30.9% vs 19.8% for men [*P* = .023] and 18.2% vs 11.8% for women [*P* = .034]), and women with cancer were more concerned about levels of sexual desire (10.2% vs 7.1%; *P* = .006). Women diagnosed < 5 years ago were more likely to report difficulty with becoming aroused (55.4% vs 31.8%; *P* = .016) and achieving orgasm (60.6% vs 28.3%; *P* < .001) and were more concerned about sexual desire (14.8% vs 7.1%; *P* = .007) and orgasmic experience (17.6% vs 7.1%; *P* = .042) than controls, but there were no differences in men.

**CONCLUSIONS:**

Self‐reports of sexual activity and functioning in older people with cancer are broadly comparable to age‐matched, cancer‐free controls. There is a need to identify the causes of sexual dissatisfaction among long‐term cancer survivors despite apparently normal levels of sexual activity and function for their age. The development of interventions addressing low sexual desire and problems with sexual functioning in women is also important and may be particularly relevant for cancer survivors after treatment. ***Cancer* 2016;122:3883–3891.** © *2016 American Cancer Society.*

## Abstract 2

Self‐reports of sexual activity and functioning in older people with cancer are broadly comparable to age‐matched, cancer‐free controls. There is a need to identify the causes of sexual dissatisfaction among long‐term cancer survivors despite apparently normal levels of sexual activity and function for their age.

## INTRODUCTION

Almost 70% of people diagnosed with cancer in the United States survive for at least 5 years,^1^ and half of people diagnosed with cancer in the United Kingdom are now expected to survive at least 10 years.^2^ Although treatment of the cancer is the primary clinical goal, ensuring the best possible quality of life after treatment is important, and this is increasingly so with high levels of survivorship. Preservation of sexual function is a key component of quality of life, yet it remains one of the “unmet needs” commonly reported by cancer survivors.^3^

Cancer is generally a disease of later life, with more than half of cases (53%) diagnosed in people aged 50 to 74 years and with more than a third (36%) diagnosed in those 75 years old or older.^4^ At older ages, sexual function also tends to decline. For example, in the Massachusetts Male Ageing Study, which followed 1085 men aged 40 to 70 years for 9 years, significant declines were observed in almost all domains of sexual function.^5^ A survey of 3005 middle‐aged and older US men and women (57‐85 years) also found that the prevalence of sexual activity declined from 73% in those aged 57 to 64 years to 26% in those aged 75 to 85 years,^6^ and similar results have been reported in the Third National Survey of Sexual Attitudes and Lifestyles (NATSAL3) in the United Kingdom, which was the first in this series of population studies to include older adults.^7^, ^8^ In the English Longitudinal Study of Ageing (ELSA), 76% of men and 53% of women older than 50 years reported being sexually active, and this indicates that sexual function is still an important aspect of life for a large proportion of older adults.^9^

A number of studies have compared sexual function before and immediately after cancer treatment^10^, ^11^ or compared one treatment modality with another,^12^ and both types have shown differences. However, there is a need for studies that include population‐based control samples. The few case‐control studies that have used validated assessments of sexual function have generally been performed in younger groups, and results have been mixed. In a comparison of 246 testicular cancer survivors (80% of whom were 18‐49 years old) and 236 ethnically matched and age‐matched controls using the Brief Male Sexual Function Inventory, the cancer survivor group reported impaired ejaculation and erection problems.^13^ However, a meta‐analysis of 28 retrospective studies and 7 prospective studies between 1975 and 2000 reported no significant deterioration of sexual function for testicular cancer survivors, although the authors acknowledged methodological limitations in many of the studies.^14^ As for women, in a US study of sexual activity comparing 50 cervical cancer survivors (mean age, 45.3 years) and 50 age‐matched controls, cancer survivors reported less sexual interest, more sexual dysfunction, and less sexual satisfaction. However, no difference in the frequency of sexual intercourse was recorded.^15^ A study in Iran comparing 71 young breast cancer survivors (mean age, 41.3 years) with 94 women without cancer (mean age, 44.2 years) found no significant differences in the prevalence of sexual dysfunction with the Female Sexual Function Index.^16^

It is important to determine whether sexual problems experienced by older cancer survivors are significantly different from those of older adults generally. One of the few studies in the older age group compared survivors of colon and rectal cancer (n = 1359) with cancer‐free controls (n = 400) and reported that male colorectal cancer survivors were slightly less sexually active than controls, whereas women reported lower enjoyment of sex. Male rectal cancer survivors also had more erectile and ejaculatory problems.^17^

The aim of the current study was, therefore, to add to the evidence base through a comparison of sexual function in cancer survivors and cancer‐free controls based on data from a population‐based sample of older adults in the United Kingdom.

## MATERIALS AND METHODS

### Study Population

Data were drawn from wave 6 (2012‐2013) of ELSA. ELSA is a longitudinal panel study of aging, health, and well‐being among men and women aged 50 years or older and living in private households in England.^18^ Of the 10,601 individuals who were interviewed in wave 6 of ELSA, 7079 (67% of those eligible) completed the paper‐based questionnaire that included measures of sexual attitudes and behavior. After the exclusion of 389 participants (5.5%) due to missing data on cancer, our final sample comprised 6690 men and women. All participants gave full informed consent to participate in the study, and ethical approval was obtained from the London Multi‐Centre Research Ethics Committee.

### Measures

Wave 6 of ELSA was the first to include questions on sexual relationships and activities. The Sexual Relationships and Activities Questionnaire (SRA‐Q) includes items taken from validated instruments,^19^, ^20^, ^21^ with minor modifications made to create versions specific for men (37 items) and women (31 items). The male and female versions of the SRA‐Q are available online.^22^ Participants completed the questionnaire in private and returned it in a sealed envelope. The SRA‐Q captures a wide range of information on attitudes to sex; the frequency of sexual activities and behaviors; problems with sexual activities and function; concerns and worries about sexual activities, sexual function, and relationships; and details about current sexual partners and satisfaction. For the purpose of our analyses, we focused on items related to sexual activities and behavior, sexual functioning, and sexual concerns and satisfaction, as summarized in Table 1.

**Table 1:** Measures of Sexual Behavior, Function, and Concerns in the Sexual Relationships and Activities Questionnaire of the English Longitudinal Study of Ageing

Item^a^	Response Options	Classification
Sexual behavior and activities (during the past month)		
How often did you think about sex?	7‐point scale: “not at all” to “more than once a day”	Thinking about sex frequently: “2‐3 times a month” or more
How many times have you had or attempted sexual intercourse (vaginal, anal, or oral)?	7‐point scale: “not at all” to “more than once a day”	Frequent sexual intercourse: “2‐3 times a month” or more
How frequently did you engage in other sexual activities (kissing, petting, or fondling)?	7‐point scale: “not at all” to “more than once a day”	Frequent kissing, petting, or fondling: “2‐3 times a month” or more
Sexual function (during the past month)		
Are you able to get or keep an erection which would be good enough for sexual activity? [men]	4‐point scale: “always able” to “never able”	Erectile difficulties: “never able” or “sometimes able”
How often did you feel sexually aroused during sexual activity? [women]	5‐point scale: “never” to “always”	Difficulty becoming sexually aroused: “never” or “much less than half the time”
When you had sexual stimulation, how difficult was it for you to reach orgasm?	5‐point scale: “impossible” to “not at all”	Difficulty achieving orgasm: “moderately difficult” to “impossible”
Sexual concerns (during the past month)		
Have you been worried or concerned by your level of sexual desire?	5‐point scale: “not at all worried or concerned” to “extremely worried or concerned”	Concerned about level of sexual desire: “moderately,” “very,” or “extremely worried or concerned”
Have you been worried or concerned by the frequency of your sexual activities?	5‐point scale: “not at all worried or concerned” to “extremely worried or concerned”	Concerned about frequency of sexual activities: “moderately,” “very,” or “extremely worried or concerned”
Have you been worried or concerned by your ability to have an erection? [men]	5‐point scale: “not at all worried or concerned” to “extremely worried or concerned”	Concerned about ability to have an erection: “moderately,” “very,” or “extremely worried or concerned”
Are you worried or concerned by your current ability to become sexually aroused? [women]	5‐point scale: “not at all worried or concerned” to “extremely worried or concerned”	Concerned about ability to become sexually aroused: “moderately,” “very,” or “extremely worried or concerned”
Have you been worried or concerned by your orgasmic experience?	5‐point scale: “not at all worried or concerned” to “extremely worried or concerned”	Concerned about orgasmic experience: “moderately,” “very,” or “extremely worried or concerned”
Sexual satisfaction (during the past three months)		
How satisfied have you been with your overall sex life?	5‐point scale: “very satisfied” to “very dissatisfied”	Dissatisfied with overall sex life: “moderately dissatisfied” or “very dissatisfied”

a All items were asked of both men and women unless otherwise specified.

Cancer diagnoses were assessed by the presentation of a list of conditions that included cancer and the following question: “Has a doctor ever told you that you have (or have had) any of the conditions on this card?” Nonmelanoma skin cancers were excluded.

Demographic information used in the current analyses included age, sex, partnership status (married/cohabiting, separated/divorced, widowed, or single/never married), and nonpension wealth quintile (a sensitive indicator of socioeconomic status in this population group^23^). Data on participant‐reported diagnoses of diabetes, coronary heart disease, arthritis, asthma, and chronic lung disease were also included, and from these data, we created a comorbidity score that indicated the number of these conditions (range, 0‐5).

### Statistical Analysis

We used weights to correct for sampling probabilities and for differential nonresponse and to calibrate back to the 2011 national census population distributions for age and sex. The weights accounted for the differential probability of being included in wave 6 of ELSA and for nonresponse to the SRA‐Q. Details can be found online.^24^ Analyses were conducted with IBM SPSS 19, with a *P* value < .05 indicating statistical significance.

Participants were categorized as those who reported a diagnosis of cancer or as controls who had never been diagnosed with cancer. This was favored over a completely healthy control group because it enabled us to determine the specific influence of a cancer diagnosis over and above other chronic diseases (which both groups might have). The 2 groups' characteristics were compared with independent *t* tests (continuous variables) and chi‐square analyses (categorical variables). Logistic regression was used to test the odds of frequent sexual activities, difficulties with sexual functioning, and sexual concerns in the cancer survivor group versus the control group. For each outcome, age‐adjusted percentages were examined with logistic regression models; we adjusted them for sampling probabilities and differential nonresponse and controlled for comorbidities. We adjusted for age and comorbid health conditions in light of the findings of a previous study in this cohort, which revealed significantly lower levels of sexual activity and poorer sexual functioning among participants who were older and in poorer health.^9^ We did not adjust for partnership status or wealth because the groups did not differ significantly on these variables.

To examine whether differences between cancer survivors and controls differed according to the time since diagnosis, analyses were repeated with stratification by the time since diagnosis (within the last 5 years vs more than 5 years earlier). The 5‐year cutoff was selected because it is a commonly used survival statistic.^25^ All analyses were performed separately for women and men because there were some differences in the male and female SRA‐Q items, and previous studies have observed notable sex differences in sexual attitudes and behavior.^6^, ^8^, ^9^, ^26^, ^27^

## RESULTS

There were 2982 men and 3708 women in the study; 220 of the men (7.4%) and 341 of the women (9.2%) had been diagnosed with cancer. Among the cancer survivors, 430 (76.6%) had been diagnosed ≥ 5 years previously. The mean time since diagnosis was 11.35 years (standard deviation, 8.90 years), and the range was 2 to 57 years (median, 9.0 years). In line with data from cancer registries,^28^ the most prevalent cancers in our sample were breast cancer, prostate cancer, colorectal cancer, and melanoma.

Characteristics of the cancer survivor and cancer‐free control groups are shown in Table 2. Cancer survivors were significantly older than controls (71.1 vs 65.9 years for men and 68.9 vs 65.2 years for women; *P* < .001 for both), and they reported more comorbid conditions (0.92 vs 0.73 for men and 0.92 vs 0.77 for women; *P* = .003 for both). The groups did not differ significantly by partnership status or wealth.

**Table 2:** Characteristics of the Cancer Survivors and Controls

	Men (n = 2982)			Women (n = 3708)		
	Cancer Survivors	Controls	*P*	Cancer Survivors	Controls	*P*
No.	220	2762		341	3367	
Age, mean (SD), y	71.14 (8.09)	65.91 (8.68)	**<.001**	68.87 (8.37)	65.18 (8.33)	**<.001**
Partnership status, % (No.)						
Married/cohabiting	80.0 (176)	77.0 (2126)	.62	60.4 (206)	64.4 (2169)	.41
Separated/divorced	9.1 (20)	9.7 (267)	—	14.7 (50)	14.4 (484)	—
Widowed	6.4 (14)	6.7 (184)	—	19.1 (65)	16.5 (557)	—
Single/never married	4.5 (10)	6.7 (185)	—	5.9 (20)	4.7 (157)	—
Wealth quintile, % (No.)						
1 (poorest)	10.6 (23)	13.1 (317)	.42	13.6 (46)	16.8 (494)	.16
2	20.7 (45)	16.8 (405)	—	16.3 (55)	19.9 (586)	—
3	19.4 (42)	21.1 (510)	—	22.2 (75)	20.9 (617)	—
4	22.1 (48)	24.2 (583)	—	25.1 (85)	21.2 (626)	—
5 (richest)	27.2 (59)	24.8 (597)	—	22.8 (77)	21.1 (623)	—
No. of comorbidities, mean (SD)	0.92 (1.01)	0.73 (0.88)	**.003**	0.92 (0.91)	0.77 (0.85)	**.003**

Abbreviation: SD, standard deviation.

Numbers may not add up to the total because of missing data for some variables. Valid percentages are shown for ease of interpretation. Bolded *P* values are significant.

Tables 3 and 4 show the age‐adjusted proportions of women and men who reported engaging in sexual activity, experiencing sexual difficulties, and having concerns about their sex lives. Approximately half of the women in both the cancer survivor and control groups reported frequent sexual intercourse (49.1% vs 50.1%; odds ratio [OR], 0.98; 95% confidence interval [CI], 0.70‐1.38). There were no differences in measures of sexual function (difficulties in becoming sexually aroused in women and erectile function in men). Women who had received a cancer diagnosis were significantly more dissatisfied with their sex lives than controls were (18.2% vs 11.8%; OR, 1.84; 95% CI, 1.05‐3.25) and expressed more concerns about their levels of sexual desire (10.2% vs 7.1%; OR, 1.83; 95% CI, 1.19‐2.81; Table 3). Similar proportions of men in the cancer survivor and control groups reported being sexually active (76.0% vs 78.5%; OR, 0.93; 95% CI, 0.66‐1.31). As in the case of women, male cancer survivors reported being significantly more dissatisfied with their sex lives than controls (30.9% vs 19.8%; OR, 1.74; 95% CI, 1.08‐2.80). Independently of diagnosis, individuals with a partner reported higher levels of sexual activity than those without a partner.

**Table 3:** Sexual Activities, Functioning, and Concerns in Cancer Survivors and Controls: Women

	Controls/Cancer Survivors, No.	Age‐Adjusted Percentages		Adjusted OR (95% CI)	*P*
		Controls	Cancer Survivors		
Sexual activity and behavior					
Any sexual activity in the past year					
All	3367/341	55.5	58.2	1.17 (0.90‐1.54)	.24
Living with a partner	2313/222	71.1	69.8	0.96 (0.68‐1.35)	.81
Not living with a partner	1054/119	25.0	30.4	1.48 (0.86‐2.56)	.16
Thinking about sex frequently	3332/339	47.2	50.8	1.17 (0.89‐1.54)	.25
Frequent sexual intercourse^a^					
All	1915/170	50.1	49.1	0.98 (0.70‐1.38)	.91
Living with a partner	1625/143	52.6	52.1	0.99 (0.68‐1.43)	.94
Not living with a partner	290/27	36.6	30.2	0.83 (0.31‐2.25)	.71
Frequent kissing, petting, or fondling^a^					
All	1914/171	67.8	69.0	0.95 (0.67‐1.36)	.78
Living with a partner	1625/144	72.3	74.2	0.98 (0.65‐1.47)	.91
Not living with a partner	289/27	42.9	37.9	0.67 (0.26‐1.74)	.41
Sexual function					
Difficulty becoming sexually aroused^b^	1392/116	31.8	31.4	0.95 (0.61‐1.47)	.81
Difficulty achieving orgasm^b^	1332/109	28.3	29.3	1.13 (0.73‐1.77)	.58
Sexual health concerns and satisfaction					
Concerned about …					
Level of sexual desire	3342/340	7.1	10.2	1.83 (1.19‐2.81)	**.006**
Frequency of sexual activities^a^	1926/171	8.0	5.4	0.57 (0.25‐1.28)	.17
Ability to become sexually aroused^b^	1384/115	7.6	6.4	0.76 (0.33‐1.76)	.52
Orgasmic experience^b^	1333/110	7.1	10.4	1.53 (0.77‐3.03)	.22
Dissatisfied with overall sex life^a^	1419/108	11.8	18.2	1.84 (1.05‐3.25)	**.034**

Abbreviations: CI, confidence interval; OR odds ratio.

The ORs are for the cancer group versus the controls (the reference group), and they have been adjusted for age and comorbidities. All analyses have been weighted for sampling probabilities and differential nonresponse. Bolded *P* values are significant.

a In participants reporting any sexual activity in the past year.

b In participants reporting any sexual activity in the past month.

**Table 4:** Sexual Activities, Functioning, and Concerns in Cancer Survivors and Controls: Men

	Controls/Cancer Survivors, No.	Age‐Adjusted Percentages		Adjusted OR (95% CI)	*P*
		Controls	Cancer Survivors		
Sexual activity and behavior					
Any sexual activity in the past year					
All	2762/220	78.5	76.0	0.93 (0.66‐1.31)	.67
Living with a partner	2254/182	81.4	77.0	0.81 (0.55‐1.20)	.29
Not living with a partner	508/38	68.5	70.2	1.35 (0.62‐2.97)	.45
Thinking about sex frequently	2749/217	81.3	76.3	0.79 (0.56‐1.11)	.18
Frequent sexual intercourse^a^					
All	2137/138	48.0	49.0	1.18 (0.81‐1.70)	.39
Living with a partner	1796/116	51.0	52.7	1.21 (0.81‐1.82)	.35
Not living with a partner	341/22	35.6	27.7	0.79 (0.28‐2.24)	.66
Frequent kissing, petting, or fondling^a^					
All	2139/138	63.7	68.1	1.20 (0.83‐1.74)	.33
Living with a partner	1799/116	68.5	74.0	1.29 (0.84‐1.96)	.25
Not living with a partner	340/22	43.4	31.9	0.62 (0.23‐1.70)	.36
Sexual function					
Erectile difficulties	2724/217	39.3	40.3	0.97 (0.69‐1.35)	.84
Difficulty achieving orgasm^b^	1536/92	13.5	13.2	0.91 (0.51‐1.65)	.76
Sexual health concerns and satisfaction					
Concerned about …					
Level of sexual desire	2758/218	13.9	11.1	0.70 (0.43‐1.13)	.14
Frequency of sexual activities^a^	2147/139	13.1	15.7	1.17 (0.72‐1.91)	.53
Ability to have an erection	2747/219	13.2	14.0	1.03 (0.68‐1.54)	.90
Orgasmic experience^b^	1520/90	10.6	11.4	0.92 (0.47‐1.79)	.80
Dissatisfied with overall sex life^a^	1442/85	19.8	30.9	1.74 (1.08‐2.80)	**.023**

Abbreviations: CI, confidence interval; OR odds ratio.

The ORs are for the cancer group versus the controls (the reference group), and they have been adjusted for age and comorbidities. All analyses have been weighted for sampling probabilities and differential nonresponse. Bolded *P* values are significant.

a In participants reporting any sexual activity in the past year.

b In participants reporting any sexual activity in the past month.

Tables 5 and 6 show results stratified by the time since diagnosis. Stratification according to the time since diagnosis indicated that differences in sexual activity and function with respect to controls were more pronounced among female cancer survivors diagnosed within the last 5 years than those diagnosed more than 5 years earlier. Levels of sexual activity did not differ, but women who had been diagnosed within the past 5 years were more likely to report difficulties with sexual function, with significantly more survivors than controls reporting difficulty in becoming sexually aroused (55.4% vs 31.8%; OR, 2.75; 95% CI, 1.21‐6.26) and achieving orgasm (60.6% vs 28.3%; OR, 4.72; 95% CI, 1.98‐11.24). However, these differences were not present in those diagnosed more than 5 years ago, and only differences in satisfaction with sex life was observed, with female cancer survivors reporting lower overall satisfaction with their sex lives (reporting dissatisfaction, 18.7% vs 11.8%; OR, 1.93; 95% CI, 1.03‐3.61).

**Table 5:** Differences in Self‐Reported Sexual Activities, Functioning, and Concerns in Cancer Survivors Diagnosed Within the Last 5 Years and Cancer Survivors Diagnosed More Than 5 Years Ago in Comparison With Controls: Women

	Controls (n = 3367), %^a^	Cancer Survivors Diagnosed < 5 y Ago (n = 63)			Cancer Survivors Diagnosed ≥ 5 y Ago (n = 278)		
		%^a^	Adjusted OR (95% CI)	*P*	%^a^	Adjusted OR (95% CI)	*P*
Sexual activity and behavior							
Any sexual activity in the past year							
All	55.5	61.0	1.11 (0.62‐1.97)	.73	57.5	1.19 (0.89‐1.61)	.25
Living with a partner	71.1	70.9	0.83 (0.42‐1.61)	.58	69.4	1.00 (0.68‐1.47)	.99
Not living with a partner	25.0	27.9	0.92 (0.18‐4.58)	.91	30.8	1.58 (0.89‐2.80)	.12
Thinking about sex frequently	47.2	48.2	0.90 (0.49‐1.63)	.72	51.5	1.25 (0.93‐1.69)	.15
Frequent sexual intercourse^b^	50.1	46.8	1.01 (0.49‐2.06)	.98	49.8	0.97 (0.66‐1.43)	.89
Frequent kissing, petting, or fondling^b^	67.8	60.1	0.65 (0.32‐1.33)	.24	71.6	1.06 (0.71‐1.58)	.78
Sexual function							
Difficulty becoming sexually aroused^c^	31.8	55.4	2.75 (1.21‐6.26)	**.016**	23.5	0.63 (0.37‐1.09)	.097
Difficulty achieving orgasm^c^	28.3	60.6	4.72 (1.98‐11.24)	**<.001**	19.1	0.65 (0.37‐1.16)	.14
Sexual health concerns and satisfaction							
Concerned about …							
Level of sexual desire	7.1	14.8	2.84 (1.34‐6.04)	**.007**	9.1	1.57 (0.94‐2.60)	.083
Frequency of sexual activities^b^	8.0	3.4	0.65 (0.12‐3.47)	.62	6.0	0.54 (0.22‐1.37)	.20
Ability to become sexually aroused^c^	7.6	6.3	0.85 (0.17‐4.14)	.84	6.4	0.73 (0.28‐1.93)	.53
Orgasmic experience^c^	7.1	17.6	3.05 (1.04‐8.93)	**.042**	8.0	1.13 (0.48‐2.67)	.77
Dissatisfied with overall sex life^b^	11.8	16.5	1.55 (0.46‐5.22)	.48	18.7	1.93 (1.03‐3.61)	**.039**

Abbreviations: CI, confidence interval; OR odds ratio.

The ORs are for the cancer survivors versus the controls (the reference group), and they have been adjusted for age and comorbidities. All analyses have been weighted for sampling probabilities and differential nonresponse. Bolded *P* values are significant.

a Adjusted for age.

b In participants reporting any sexual activity in the past year.

c In participants reporting any sexual activity in the past month.

**Table 6:** Differences in Self‐Reported Sexual Activities, Functioning, and Concerns in Cancer Survivors Diagnosed Within the Last 5 Years and Cancer Survivors Diagnosed More Than 5 Years Ago in Comparison With Controls: Men

	Controls (n = 2762), %^a^	Cancer Survivors Diagnosed < 5 y Ago (n = 68)			Cancer Survivors Diagnosed ≥ 5 y Ago (n = 152)		
		%^a^	Adjusted OR (95% CI)	*P*	%^a^	Adjusted OR (95% CI)	*P*
Sexual activity and behavior							
Any sexual activity in the past year							
All	78.5	74.3	0.81 (0.44‐1.50)	.50	76.7	0.98 (0.65‐1.47)	.92
Living with a partner	81.4	73.3	0.59 (0.30‐1.17)	.13	78.7	0.92 (0.58‐1.45)	.72
Not living with a partner	68.5	79.5	2.11 (0.51‐8.74)	.31	66.0	1.11 (0.44‐2.81)	.82
Thinking about sex frequently	81.3	73.6	0.66 (0.36‐1.21)	.18	77.5	0.85 (0.56‐1.27)	.42
Frequent sexual intercourse^b^	48.0	39.5	0.77 (0.40‐1.47)	.42	53.4	1.44 (0.92‐2.23)	.11
Frequent kissing, petting, or fondling^b^	63.7	76.9	1.85 (0.92‐3.73)	.087	64.1	1.01 (0.65‐1.55)	.98
Sexual function							
Erectile difficulties	39.3	38.3	0.87 (0.48‐1.55)	.63	41.2	1.01 (0.68‐1.52)	.95
Difficulty achieving orgasm^c^	13.5	18.3	1.27 (0.48‐3.39)	.63	10.7	0.78 (0.38‐1.61)	.51
Sexual health concerns and satisfaction							
Concerned about …							
Level of sexual desire	13.9	11.3	0.68 (0.29‐1.56)	.36	11.0	0.71 (0.40‐1.25)	.24
Frequency of sexual activities^b^	13.1	20.5	1.89 (0.91‐3.89)	.090	13.5	0.87 (0.46‐1.67)	.68
Ability to have an erection	13.2	17.2	1.35 (0.70‐2.60)	.38	12.6	0.90 (0.54‐1.48)	.67
Orgasmic experience^c^	10.6	11.1	0.90 (0.27‐3.00)	.87	11.5	0.92 (0.42‐2.02)	.84
Dissatisfied with overall sex life^b^	19.8	26.3	1.34 (0.55‐3.28)	.53	32.9	1.92 (1.11‐3.34)	**.020**

Abbreviations: CI, confidence interval; OR odds ratio.

The ORs are for the cancer survivors versus the controls (the reference group), and they have been adjusted for age and comorbidities. All analyses have been weighted for sampling probabilities and differential nonresponse. Bolded *P* values are significant.

a Adjusted for age.

b In participants reporting any sexual activity in the past year.

c In participants reporting any sexual activity in the past month.

There were no significant differences in any aspect of sexuality between male cancer survivors diagnosed less than 5 years ago and male cancer‐free controls. However, the greater dissatisfaction with sex life reported in the complete sample of male cancer survivors was limited to men diagnosed more than 5 years ago (32.9% vs 19.8%; OR, 1.92; 95% CI, 1.11‐3.34).

## DISCUSSION

This is the first analysis of cohort data to compare sexual behavior and concerns between cancer survivors and controls from the same population‐based study with a standardized measure. The main finding is that cancer survivors do not differ from cancer‐free controls in whether they are sexually active or in the frequency of different types of sexual activity. In the shorter term (<5 years after the diagnosis), female cancer survivors were more likely to experience sexual dysfunction. However, more than 5 years after the diagnosis, the only significant difference was that cancer survivors reported more dissatisfaction with their sex lives. There were no differences between male cancer survivors and controls in erectile difficulties or problems achieving orgasm. These findings are important because an extremely high proportion of cancer patients will become long‐term survivors, and a clear picture of life after cancer is required.

To our knowledge, just one other study has examined differences in sexual function in older cancer survivors; it compared 1359 colorectal cancer survivors and a small sample of age‐matched controls.^9^ In this sample, sexual function was reduced in both men and women in the cancer survivor group. However, in men, there was no significant difference in sexual enjoyment. The time since diagnosis was not reported in this study,^17^ and the use of a small normative population was a limitation. However, it is likely that the nature of treatments for certain cancers (eg, colorectal, prostate, or gynecological cancers) has a more significant impact on sexual function in the short term.

Our results show less striking differences between cancer survivors and controls than some previous studies.^10^, ^11^, ^12^, ^13^, ^15^ This might be due to 2 factors. First, the focus of much previous research has been on younger survivors, who are in such a minority and among whom concerns related to sexuality may differ (eg, reproductive issues). Because cancer is a disease that occurs predominantly at older ages, the results of these studies may not be generalizable to older cancer survivors. Our study focused on those older than 50 years, who receive more than 90% of the cancer diagnoses in the UK population^4^; data were drawn from a population‐based cohort known to be broadly representative of this age group.^18^ Second, in some previous studies, there has been an absence of age‐relevant comparison groups. Because levels of sexual activity decline with age, cancer survivors may attribute any changes to their health condition rather than the passage of time. Our study suggested that in longer term survivorship, there are few differences between cancer survivors and controls. It may be that aging causes controls to “catch up” to cancer survivors in terms of sexuality. Cancer survivors may experience an accelerated decline in sexuality around the time of diagnosis, whereas changes happen more gradually over time in individuals who remain cancer‐free, but it seems that both groups ultimately reach similar levels of sexual activity and functioning. To extend the existing literature, future research should examine differences between specific cancer sites and age‐matched controls in older adults and include long‐term follow‐up.

Although we found few differences in long‐term cancer survivors, women in our study who were diagnosed <5 years ago reported more sexual dysfunction. In a recent large qualitative study of 1514 cancer survivors with mixed sites, physical symptoms (including sexual dysfunction) were the most commonly reported unmet care needs (38.2% of the participants). Interestingly, in this study, older cancer survivors were more likely than younger participants to report unmet needs in the physical domain.^28^ Our results suggest that a large proportion of cancer survivors remain sexually active, whether they live with partners or not, so addressing aspects of sexual dysfunction (particularly in women soon after their diagnosis) is important.

Our study indicated that long‐term cancer survivors were less satisfied with their sex lives, despite levels of arousal, sexual activity, and sexual function broadly similar to those of controls. There is currently a lack of evidence showing that older cancer survivors do dramatically differ in terms of sexual function. However, the reasons behind the significantly lower satisfaction with sex lives reported by both male and female cancer survivors in our study are an area for further research because this may direct the development and nature of rehabilitation packages. There is a need to develop such packages to arm clinicians with the tools to address sexual function after cancer treatment. In a qualitative study, colorectal cancer survivors and their partners both emphasized the importance of receiving sexual health care and the need to be informed about treatment options for any problems with sexual function that could arise.^3^ However, in clinical consultations, they had not felt able to raise the issue of sexual health because they saw it as a side issue to the main focus of clinical consultations: survival. In the same study, health professionals reported similar concerns about the appropriateness of discussing sex when survival was the primary concern, and they hesitated to raise the issue for fear of making patients and their partners feel uncomfortable. In some cases, health professionals reported feeling that discussing sex would be irrelevant, particularly if patients were older, female, or single.^3^ However, data from NATSAL3 and from the current study suggest that such assumptions are not necessarily correct.^7^

This study benefited from a detailed standard measure of sexual functioning and the availability of information on comorbid health conditions as well as a control population recruited on a similar basis. However, there were a number of limitations. Cancer data were self‐reported, but previous studies have shown high agreement between self‐reported cancer diagnoses and medical record validation in population‐based samples.^29^, ^30^, ^31^ The sample of cancer survivors was not large enough for an analysis by cancer site, and there may be differences between sites. We did not have detailed information on the date of diagnosis (or the type of treatment). Sensitivity analyses in which cancer survivors were stratified by the time since diagnosis suggested that differences were more pronounced among those diagnosed within the last 5 years, but small sample sizes meant that these analyses lacked statistical power. We performed a large number of statistical tests, and it is possible that adjustments for multiple comparisons might reduce the number of significant findings.

In conclusion, on the whole, our results are generally encouraging: they show that older people with cancer do not experience greater problems with sexual activity or functioning than age‐matched, cancer‐free controls. A diagnosis of cancer does not seem to affect whether or not people have sex, how often they have sex, what they do when they have sex, and (in the case of men) their sexual function. Compared with age‐matched controls, women who had been diagnosed with cancer within the past 5 years were just as likely to be sexually active, although they were more likely to report problems with arousal. Following the 5 years after the diagnosis, the only difference was greater dissatisfaction with their sex lives. Male survivors did not report any more significant sexual problems than their age‐matched counterparts, but they were more dissatisfied with their sex lives.

Further research is required to address sexual difficulties in women in the immediate postdiagnosis phase. There is currently very little available to help women deal with low sexual desire. This is especially the case if they have a history of certain breast cancers or certain gynecological cancers and in light of the fact that systemic hormone replacement therapy is generally not recommended in women, regardless of their cancer history.

Future studies should also seek to establish why dissatisfaction with one's sex life is higher among long‐term cancer survivors even though their sexual function is similar to that of the general population. On the basis of the results of the current study and other studies in both cancer survivors and the general population, it is evident that there is a need to identify interventions to enhance sexual health in aging men and women. In the meantime, better advice on normal changes in sexual activity and functioning with aging could help to address the discrepancy between normative sexual behavior and lower sexual satisfaction in cancer survivors.

## FUNDING SUPPORT

This work was supported by Cancer Research UK (C1418/A14133).

## CONFLICT OF INTEREST DISCLOSURES

The authors made no disclosures.

## AUTHOR CONTRIBUTIONS

Sarah E. Jackson: Conception and design of the study, analysis and interpretation of the data, writing of the manuscript, and approval of the final manuscript. Jane Wardle: Conception and design of the study, analysis and interpretation of the data, writing of the manuscript, and approval of the final manuscript. Andrew Steptoe: Conception and design of the study, analysis and interpretation of the data, writing of the manuscript, and approval of the final manuscript. Abigail Fisher: Conception and design of the study, analysis and interpretation of the data, writing of the manuscript, and approval of the final manuscript.
